# High extracellular polymeric substance production and biofilm-forming capacity of *Ralstonia pickettii* isolates from ISS potable water

**DOI:** 10.1128/spectrum.02913-25

**Published:** 2026-02-05

**Authors:** Takehiko Kenzaka, Tomoaki Ichijo, Takashi Yamazaki

**Affiliations:** 1 Faculty of Science and Engineering, Setsunan University 47731, Neyagawa, Osaka, Japan; 2 Faculty of Pharmacy, Osaka Ohtani University 38308 , Tondabayashi, Osaka, Japan; 3 Faculty of Health and Nutrition, Osaka Shoin Women’s University, Higashi-Osaka, Osaka, Japan; 4 Graduate School of Human Sciences, Osaka Shoin Women’s University, Higashi-Osaka, Osaka, Japan; 5 Department of Interdisciplinary Space Science, Institute of Space and Astronautical Science, Japan Aerospace Exploration Agency (JAXA) 83988 , Sagamihara, Kanagawa, Japan; 6 Space Environment Utilization Center, Human Spaceflight Technology Directorate, JAXA 88309 , Tsukuba, Ibaraki, Japan; 7 Space and Astronautical Science, Graduate Institute for Advanced Studies, SOKENDAI 13177, Hayama, Kanagawa, Japan; 8 General Medical Education and Research Center, Teikyo University, Itabashi, Tokyo, Japan; Connecticut Agricultural Experiment Station, New Haven, Connecticut, USA

**Keywords:** *Ralstonia*, extracellular polymeric substance, potable water, space

## Abstract

The potable water dispenser (PWD) system plays a critical role as a source of drinking water for astronauts on the International Space Station (ISS). In this study, we examined the bioburden in the potable water produced by the PWD. The amount of extracellular polymeric substances (EPSs) in the PWD water was approximately 19 or 55 times greater than the bacterial count, and the EPS biomass accounted for approximately 24% or 86% of the bacterial biomass. *Ralstonia pickettii* consistently comprised approximately 70% or 80% of the bacteria for 3 years. Under simulated microgravity conditions, the isolated *R. pickettii* strains exhibited higher cell and EPS concentrations and higher total volume concentrations (average volume multiplied by concentration) of cell and EPS than under 1G conditions, whereas the average cell volume was smaller and the average EPS volume was larger. The ISS isolates showed higher EPS production and biofilm-formation abilities than terrestrial strains under nutrient-rich conditions and possessed high biofilm-formation ability comparable to those of terrestrial strains under nutrient-poor conditions. The ability of *R. pickettii* to produce EPS may play a crucial role in its adaptation to the water environment on the ISS.

**IMPORTANCE:**

In space habitation environments, the use of recycled water is indispensable, and ensuring its microbiological safety is essential. In this study, we elucidated the microbiological characteristics of water from the potable water dispenser (PWD) on the International Space Station (ISS). Our findings revealed that bacteria of the *Ralstonia pickettii* are the predominant species in PWD water and that extracellular polymeric substances (EPSs) constitute a large proportion of the biomass. Furthermore, the isolated *R. pickettii* was shown to possess high EPS production ability and strong biofilm-forming capacity. Since EPS plays a crucial role in biofilm formation, these abilities may be important factors enabling *R. pickettii* to adapt to the water environment of the ISS.

## INTRODUCTION

During long-term stays in manned space facilities, it is extremely important to maintain potable water meant for the crew’s consumption in a hygienic condition and ensure its microbiological safety when consumed in order to maintain their health. The potable water currently used on the International Space Station (ISS) is produced by the ISS water processor assembly (WPA). This unit provides recycled water produced by combining the condensation of air humidity with sweat and urine distillate, which is highly nutritious (1).

The ISS potable water dispenser (PWD) is positioned downstream of the ISS WPA. In a previous study, the bacterial abundance in PWD water was quantified using biological particle counters, which are based on fluorescence intensity of riboflavin contained in cells (2). The results showed that the maximum detected bacterial concentration was approximately 10^5^ cells mL^−1^. Furthermore, a comprehensive analysis of the microbiota in the ISS potable water revealed that *Ralstonia pickettii* accounted for more than 70% of the microbial load (2). These results indicate that the ISS WPA is not able to completely remove bacteria during potable water production, although the water is sufficiently treated before being used as potable water.

Therefore, there is a concern regarding the risk of biofilm formation in ISS potable water supply systems. Biofilm formation can cause contamination problems and loss of function in critical spacecraft systems, and it is believed that the effects of microgravity will further aggravate the situation (3). In addition, biofilms can significantly affect the downstream components of various water supply systems, particularly in industrial or scientific settings, causing several problems. In order to prevent biofilm formation throughout the ISS WPA, it is necessary to manage everything from the waste tank itself (4). In addition to negatively affecting the integrity and function of spacecraft materials, biofilm formation can increase antibiotic resistance, which can negatively affect human health (5).

A detailed analysis of the bacteria in the PWD water revealed traces of biofilms. One reason for this is that the ISS potable water contained a large amount of dissolved organic carbon. The microbial particle counter is equipped with deep ultraviolet (UV) light devices that emit light at 185 and 254 nm to intensify the excitation light of riboflavin. The 185 nm deep UV light device efficiently degrades the dissolved organic carbon in the ISS potable water (2). In fact, when we assessed water from the PWD, we found that the fluorescent substances derived from the dissolved organic carbon in the potable water were efficiently removed by the 185 nm UV light. The efficient degradation of dissolved organic carbon by the 185 nm deep UV light irradiation device indirectly indicates that a large amount of extracellular polymeric substances (EPSs) derived from the biofilm formed in the device is dissolved in potable water. When ISS potable water samples were examined under a fluorescence microscope, groups of bacteria wrapped in biofilm-like membranes were observed. It is believed that these detached from the biofilm formed in the PWD and flowed out along with the potable water.

In this study, the EPS in PWD water was directly observed using microscopic methods. After confirming the temporal changes in the bacterial community structure of water from the PWD and the dominance of *R. pickettii*, the EPS production and biofilm-formation abilities of the isolated *R. pickettii* were compared with those of strains isolated on Earth.

## RESULTS

### Bacterial communities in PWD water

**Fig 1:**
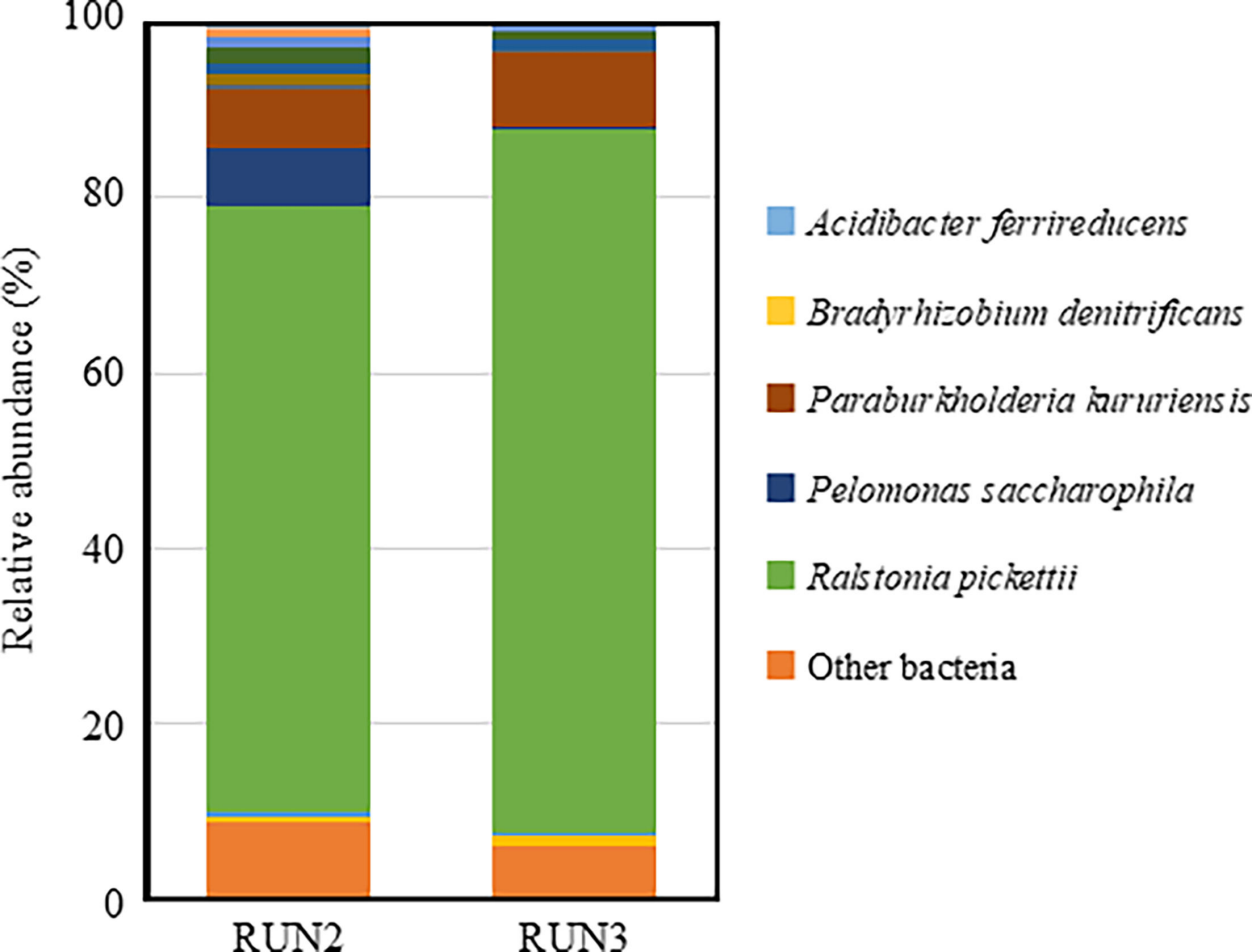
Relative abundance of bacterial taxa in water samples collected from the ISS potable water dispenser, based on 16S rRNA gene amplicon sequencing. A total of 12,783 (RUN2) and 12,474 (RUN3) reads were analyzed.

Bacterial community structures were analyzed by targeting nearly full-length 16S rRNA genes (V1–V9 region) using the Oxford Nanopore Technologies MinION platform. Water samples were collected from a PWD system and analyzed in two separate sampling runs (RUN2 and RUN3). A total of 12,783 and 12,474 reads were obtained from RUN2 and RUN3, respectively. At the species level, the dominant bacterial taxa were *R. pickettii* and *Paraburkholderia kururiensis* (Fig. 1). In RUN2, *R. pickettii* and *P. kururiensis* accounted for 69.1% and 6.8% of the total community, respectively. Similarly, in RUN3, *R. pickettii* and *P. kururiensis* accounted for 80.2% and 8.4%, respectively. Overall, *R. pickettii* was the predominant bacterial species in both samples. The bacterial community structures in RUN2 and RUN3 were similar, indicating consistent microbial profiles for 3 years, including a previous study (2).

### Cell and EPS biomass in PWD water

**Fig 2:**
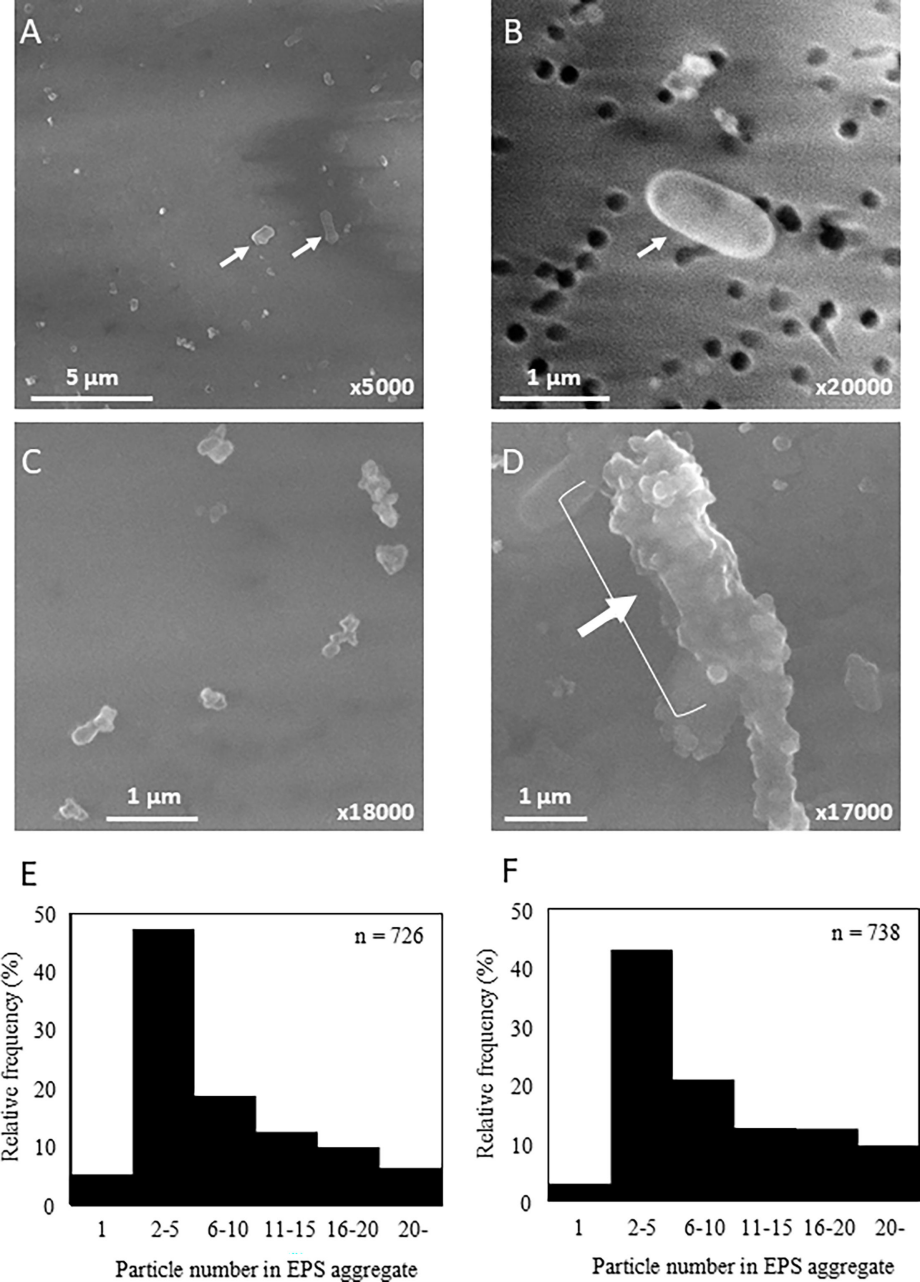
Scanning electron microscopy images of cells and extracellular polymeric substances (EPSs) from ISS PWD water. EPSs were more abundant than cells in PWD water (**A**). Single cell (**B**), aggregated EPSs (**C**), and many EPSs attached to cells (**D**) were observed. Arrows indicate cells. Distribution of particle number in EPS aggregates derived from PWD water in RUN2 (**E**) and RUN3 (**F**) was summarized.

After PWD water samples were collected through a 0.2 μm filter, scanning electron microscopy (SEM) revealed numerous EPS particles, in addition to bacterial cells in PWD water (Fig. 2A through D). EPS particles, which were much smaller than cells, were present alone, in aggregates, or attached to cells. The numbers of EPS particles in RUN2 and RUN3 samples were 8.2 × 10^5^ and 7.2 × 10^6^ particles mL^−1^, respectively, approximately 19 and 55 times (average 37 times) greater than the number of bacterial cells in RUN2 and RUN3 (4.3 × 10^4^ and 1.3 × 10^5^ cells mL^−1^), respectively (Table 1).

**TABLE 1:** Biomass of bacterial components in PWD water

Component		Concentration (particles or cells mL^−1^)	Size (μm)	Volume (μm^3^)	Total volume concentration (μm^3^ mL^−1^)
EPS	RUN2 RUN3	8.2 × 10^5^ 7.2 × 10^6^	0.18 ± 0.03^*b*^ 0.18 ± 0.04	0.0032 ± 0.0013 0.0033 ± 0.0022	2.6 ± 1.1 × 10^3^ 2.4 ± 1.6 × 10^4^
Cell	RUN2	4.3 × 10^4*a*^	1.35 ± 0.47^*c*^ 0.50 ± 0.13^*d*^	0.26 ± 0.17	1.1 ± 0.7 × 10^4^
	RUN3	1.3 × 10^5^	1.13 ± 0.47 0.49 ± 0.13	0.22 ± 0.18	2.8 ± 2.3 × 10^4^
EPS/cell^*e*^	RUN2 RUN3	19 55	0.19 ± 0.00 0.22 ± 0.00	0.012 ± 0.00 0.015 ± 0.00	0.24 ± 0.00 0.86 ± 0.01

^*a*^ Cell concentration was determined using fluorescence microscopy. EPS concentration, size, and volume of EPSs (*n* = 108 for RUN2 and *n* = 106 for RUN3), and cells (*n* = 105 for RUN2 and *n* = 108 for RUN3) were determined using scanning electron microscopy. Total volume concentration was calculated by multiplying the volume by the concentration. Values are presented as mean ± standard deviation.

^*b*^ Diameter.

^*c*^ Length.

^*d*^ Width.

^*e*^ Value in EPS was divided by value in cell.

The diameter of EPS particles was approximately 0.2 μm, which was the pore size of the filter, with most of them forming aggregates (Fig. 2E and F). Most EPS particles existed as two or more aggregates, with many forming 20 or more aggregates. Although the individual EPSs were approximately 0.2 μm in size, they formed aggregates or were attached to cells and could be collected through the 0.2 μm filter. The volume of individual EPS was approximately 1.2% and 1.5% that of a cell in RUN2 and RUN3, respectively (Table 1), and the total volume concentration (average volume multiplied by concentration) of EPS in PWD water was 2.6 × 10^3^ and 2.4 × 10⁴ μm³ mL⁻¹ in RUN2 and RUN3, respectively. They constituted 24% and 86% (average 55%) of the total volume concentration of cell in RUN2 and RUN3, respectively, owing to the higher number of particles. This indicates that EPSs are a very important component of biomass in PWD water.

### Cell and EPS biomass in culture of ISS isolate

**Fig 3:**
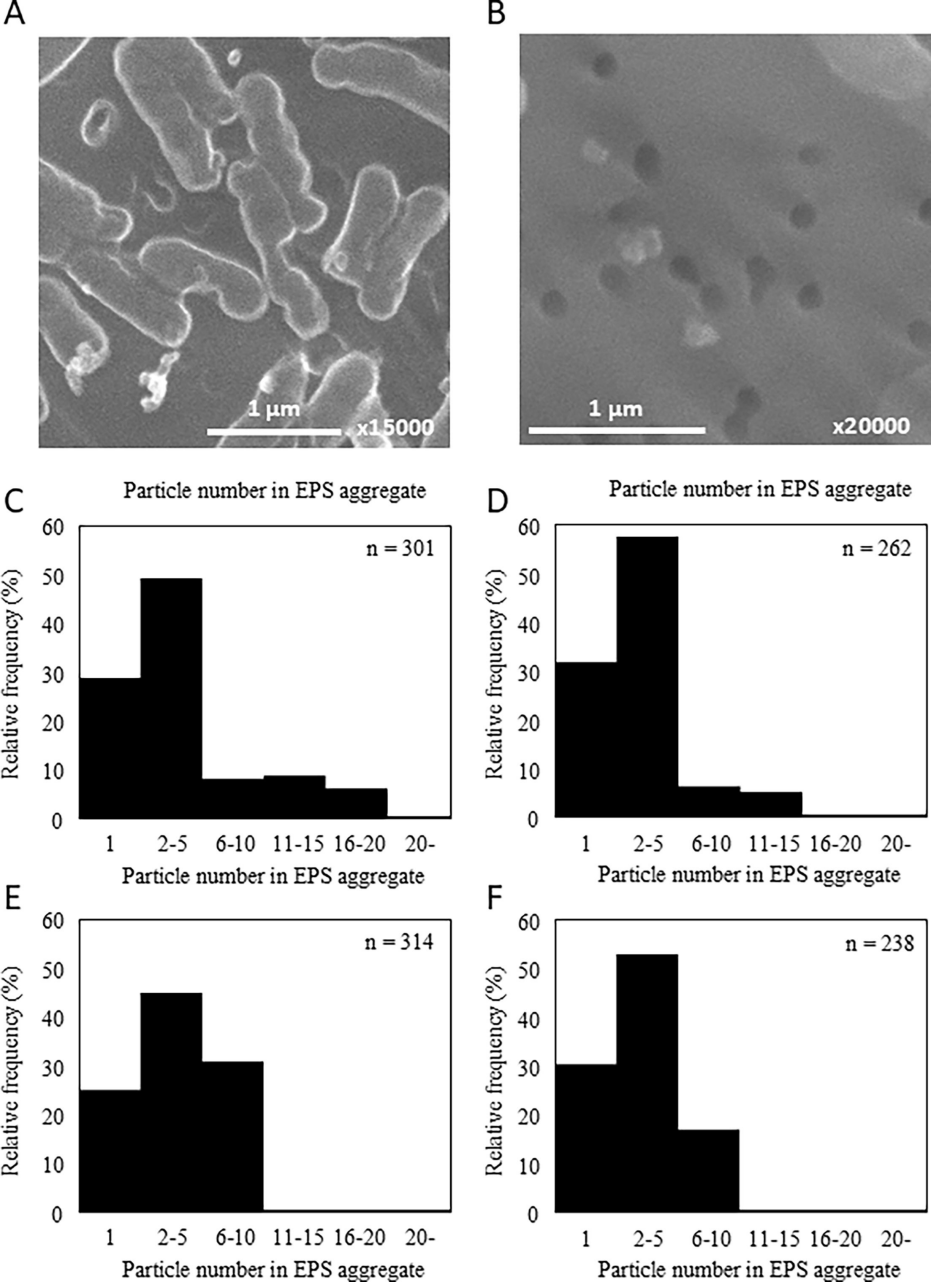
EPSs were observed in the culture of *R. pickettii* isolate ISS25 (**A and B**). Distribution of particle number in EPS aggregates derived from *R. pickettii* isolate ISS25 under 1G (**C**), *R. pickettii* isolate ISS25 under SMG (**D**), NBRC111592 under 1G (**E**), and NBRC111592 under SMG (**F**).

More than 70% of the bacteria in PWD water on the ISS were identified as *R. pickettii* (Fig. 1). The *R. pickettii* strains (ISS25, ISS26, ISS27, and ISS28) isolated from PWD water and the NBRC 111592 strain isolated from a ground aquatic environment were cultured in Mueller–Hinton (MH) broth under both 1G and simulated microgravity (SMG) conditions, and the cells and EPS biomass were analyzed (Tables 2 and 3). EPS was observed in all ISS strains (Fig. 3A and B). As the diameter of the EPS particles was approximately 0.2 μm and thus small enough to pass through a 0.2 μm filter, both EPS and cells present in the MH broth were collected using a 0.02 μm filter and subsequently analyzed using fluorescence microscopy and SEM.

**TABLE 2:** Biomass of bacterial cell in *Ralstonia pickettii* isolates^*a*^

Component	Gravity and strain	Number (cells mL^−1^)	Length or width (μm)	Volume (μm^3^)	Total volume concentration (μm^3^ mL^−1^)
Cell	ISS25 1G	5.4 ± 1.9 × 10^8^	1.56 ± 0.31^*b*^	0.32 ± 0.11	1.6 ± 0.6 × 10^8^
			0.58 ± 0.10^*c*^		
	SMG	1.2 ± 0.6 × 10^9^**	1.39 ± 0.29***	0.20 ± 0.08***	2.4 ± 0.9 × 10^8^***
			0.45 ± 0.07**		
	Ratio^*d*^	2.40	0.89^*b*^/0.77^*c*^	0.62	1.49
					
	ISS26 1G	7.1 ± 0.6 × 10^8^	1.50 ± 0.25	0.22 ± 0.08	1.6 ± 0.6 × 10^8^
			0.45 ± 0.07		
	SMG	1.4 ± 0.0 × 10^9***^	1.45 ± 0.22	0.20 ± 0.08*	2.7 ± 1.0 × 10^8***^
			0.44 ± 0.08		
	Ratio	1.91	0.97/0.97	0.91	1.74
					
	ISS27 1G	9.4 ± 0.9 × 10^8^	1.44 ± 0.27	0.26 ± 0.12	2.4 ± 1.1 × 10^8^
			0.50 ± 0.09		
	SMG	1.4 ± 0.0 × 10^9^***	1.38 ± 0.26	0.22 ± 0.08**	3.1 ± 1.1 × 10^8^***
			0.47 ± 0.08*		
	Ratio	1.51	0.96/0.96	0.86	1.29
					
	ISS28 1G	9.4 ± 0.5 × 10^8^	1.53 ± 0.28	0.29 ± 0.13	2.8 ± 1.2 × 10^8^
			0.51 ± 0.09		
	SMG	1.2 ± 0.1 × 10^9^***	1.48 ± 0.33	0.26 ± 0.12*	3.2 ± 1.4×10^8^**
			0.49 ± 0.09*		
	Ratio	1.29	0.97/0.96	0.89	1.14
					
	NBRC^*e*^ 1G	4.4 ± 4.2 × 10^8^	1.26 ± 0.31	0.21 ± 0.10	9.1 ± 4.3 × 10^7^
			0.48 ± 0.08		
	SMG	1.2±0.2 × 10^9^***	1.18 ± 0.26*	0.15 ± 0.06***	1.8 ± 0.8 × 10^8^***
			0.43 ± 0.06***		
	Ratio	2.73	0.93/0.89	0.74	2.01
*P* value^*f*^	Gravity	0.003	0.026^*b*^/0.095^*c*^	0.042	0.003
	Strain	0.213	0.008/0.340	0.119	0.007

^*a*^ Cell concentration was determined using fluorescence microscopy. The size and volume of the cells were determined using scanning electron microscopy. Total volume concentration was calculated by multiplying the volume by the concentration. Values are presented as mean ± standard deviation. An independent sample *t*-test was used to compare the values between 1G and SMG conditions. *,* P *< 0.05; **,* P *< 0.01; ***,* P *< 0.001.

^*b*^ Length.

^*c*^ Width.

^*d*^ Ratio of SMG and 1G values.

^*e*^ *R. pickettii* NBRC 111592.

^*f*^ *P* value in paired sample *t*-test between 1G and SMG or in ANOVA for between strains.

**TABLE 3:** Biomass of bacterial EPS in *Ralstonia pickettii* isolates^*a*^

Component	Gravity and strain	Number (particles mL^−1^)	Diameter (μm)	Volume (μm^3^)	Total volume concentration (μm^3^ mL^−1^)
EPS	ISS25 1G	1.0 ± 0.8 × 10^9^	0.14 ± 0.03	0.0016 ± 0.0012	1.8 ± 1.2 × 10^6^
	SMG	2.1 ± 1.6 × 10^9^***	0.16 ± 0.04**	0.0028 ± 0.0026***	5.4 ± 5.4 × 10^6^***
	Ratio^*b*^	2.05	1.13	1.54	3.17
	ISS26 1G	1.1 ± 0.5 × 10^9^	0.15 ± 0.03	0.0019 ± 0.0011	2.2 ± 1.3 × 10^6^
	SMG	1.3 ± 0.9 × 10^9^	0.17 ± 0.04***	0.0028 ± 0.0018***	3.6 ± 2.3 × 10^6^***
	Ratio	1.11	1.14	1.51	1.68
	ISS27 1G	9.7 ± 4.4 × 10^8^	0.15 ± 0.03	0.0018 ± 0.0010	1.7 ± 1.0 × 10^6^
	SMG	1.1 ± 0.6 × 10^9^*	0.16 ± 0.04*	0.0022 ± 0.0014*	2.5 ± 1.6 × 10^6^***
	Ratio	1.15	1.06	1.26	1.46
	ISS28 1G	9.5 ± 0.5 × 10^8^	0.16 ± 0.03	0.0024 ± 0.0011	2.2 ± 1.0 × 10^6^
	SMG	1.2 ± 0.7 × 10^9^**	0.19 ± 0.05***	0.0048 ± 0.0043***	5.6 ± 5.0 × 10^6^***
	Ratio	1.24	1.21	2.03	2.50
	NBRC^*c*^ 1G	1.2 ± 0.9 × 10^9^	0.14 ± 0.03	0.0018 ± 0.0013	2.1 ± 1.6 × 10^6^
	SMG	5.7 ± 4.1 × 10^8^***	0.14 ± 0.03	0.0015 ± 0.0010	8.8 ± 5.8 × 10^5^***
	Ratio	0.48	0.97	0.87	0.41
*P* value^*d*^	Gravity Strain	0.526 0.640	0.066 0.110	0.102 0.234	0.149 0.504

^*a*^ Concentration, size, and volume of the cells were determined using scanning electron microscopy. Total volume concentration was calculated by multiplying the volume by the concentration. Values are presented as mean ± standard deviation. Independent sample *t*-test was used to compare the values between 1G and SMG conditions. *,* P *< 0.05; **, *P *< 0.01; ***,* P *< 0.001.

^*b*^ Ratio of SMG and 1G values.

^*c*^ *R. pickettii* NBRC 111592.

^*d*^ *P* value in paired sample *t*-test between 1G and SMG or in ANOVA for between strains.

All *R. pickettii* strains yielded significantly higher concentrations of cells under SMG than under 1G (*P* = 0.003) (Table 2 and Fig. S1). The length and width of most cells tended to be lower under SMG than under 1G. Consequently, under SMG, the average volume of cells was lower, whereas the total volume concentration of cells was significantly higher than those under 1G across all strains (*P* = 0.042 for average volume, *P* = 0.003 for total volume concentration). The response to SMG varied among strains (overall effect: *P* = 0.007), and the increase in total volume concentration in NBRC 111592 was greater than that observed in the other ISS strains (*P* < 0.05 for ISS25 and ISS26, *P* < 0.01 for ISS 27 and 28).

In contrast, the concentration of EPS under SMG was generally higher than that under 1G for ISS strains (*P* < 0.05 for ISS25, ISS27, and ISS28; *P* = 0.069 for ISS26) but lower for NBRC 111592 (*P* < 0.001) (Table 3). In the ISS strains, the diameter of the EPS was significantly higher under SMG conditions than under 1G conditions, whereas no significant difference was observed for the NBRC 111592 strain between the 1G and SMG conditions (*P* = 0.41). Among ISS strains, the average EPS volume and total EPS volume concentration were significantly elevated under SMG relative to 1G, whereas NBRC 111592 exhibited a significantly lower total volume concentration of EPS under SMG than under 1G. The NBRC 102503T strain produced significantly less EPS than the other strains; therefore, it was not analyzed by SEM.

Overall, the size and volume characteristics of cells and EPS in the ISS strains were consistent with those observed in PWD water, as shown in Tables 1 to 3. The EPS-to-cell biomass ratio is summarized in Table S1. In all strains under both SMG and 1G, the total volume concentration of EPS represented less than 2.4% of the total volume concentration of the cells, whereas in PWD water, EPS represented 24% and 86% of the total cell volume concentration. As the PWD possesses a 0.2 µm particulate/point-of-use filter, it is conceivable that smaller EPS can more easily pass through the filter, allowing the cells to adhere more effectively and remain within the PWD.

Aggregates frequently consisted of two to five EPS particles under both 1G and SMG conditions for both ISS25 and NBRC 111592 strains (Fig. 3C through F). However, approximately 25%–31% of the particles existed as single particles, and aggregates with 11 or more particles were rare. These observations differed from those made in PWD water (Fig. 2E and F). In PWD water, non-aggregating particles with less than 0.2 μm range might pass through the 0.2 μm filter.

### Component of EPS

**Fig 4:**
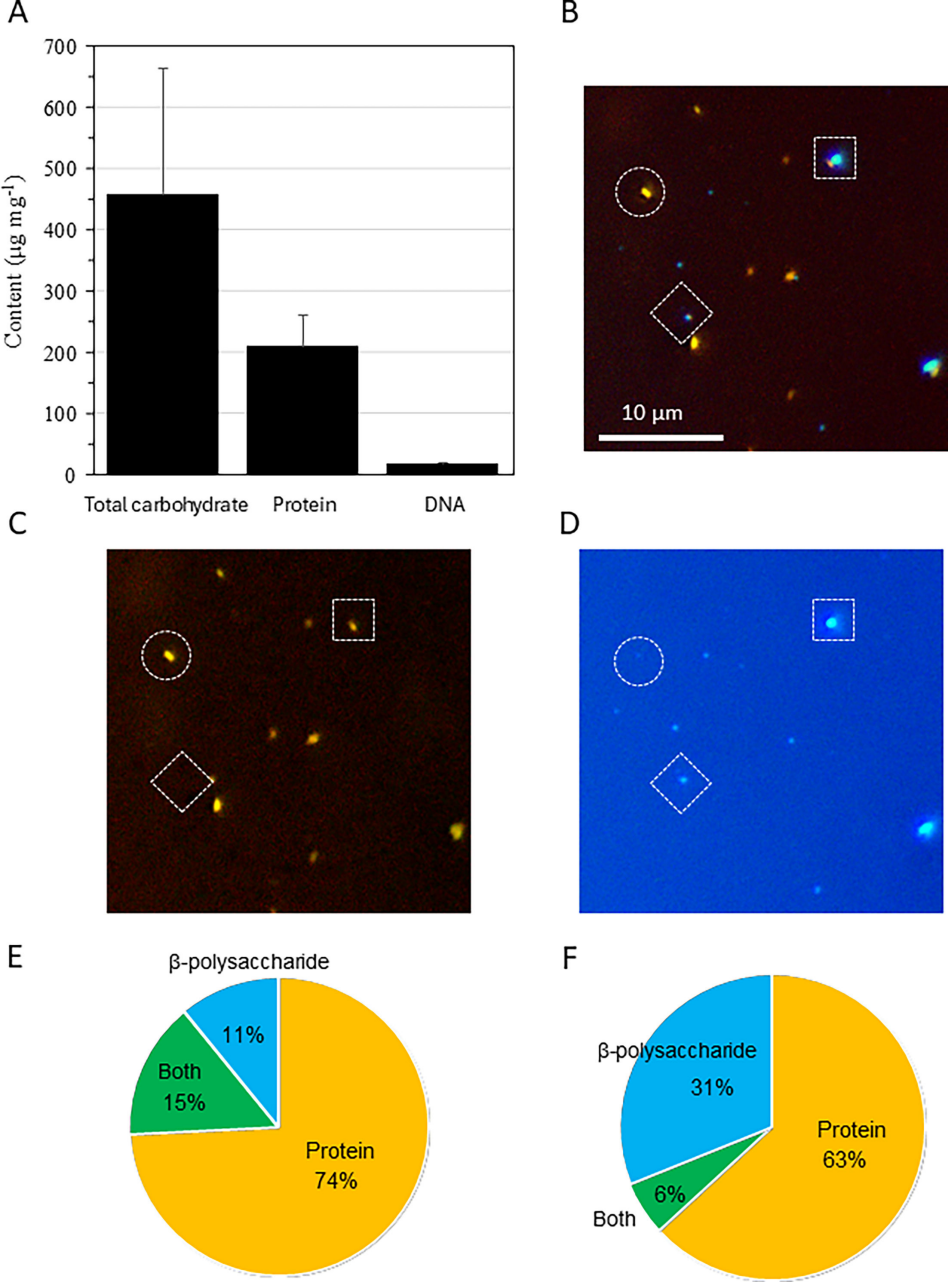
Biochemical compositions of EPSs from *R. pickettii* ISS isolates and PWD water. Biochemical compositions of EPS from *R. pickettii* ISS25, ISS26, ISS27, and ISS28 are presented as mean ± standard deviation (**A**). Cells and EPSs in PWD water were labeled by fluorescent dyes Qubit Protein Reagent and Calcoﬂuor White, and the merged fluorescent image was constructed (**B**, both; **C**, protein; **D**, β-polysaccharide). EPSs with only protein signal (dashed circles), only β-polysaccharide signal (dashed diamonds), and both protein and β-polysaccharide signals (dashed squares) were observed. The composition of the culture of *R. pickettii* isolate ISS25 in MH broth under SMG (**E**, *n* = 296) and EPS in the PWD water (**F**, *n* = 795) is summarized.

Whole EPS was extracted from the culture of four ISS strains, and compositional analysis revealed that it consisted of approximately 45% carbohydrates, 21% proteins, and 2% DNA (Fig. 4A). The whole-extract composition was subsequently compared with results from fluorescent staining of EPS particles. Double staining with Qubit Protein Reagent and 4′,6-diamidino-2-phenylindol (DAPI) in the supernatant of the ISS25 strain culture and PWD water confirmed that EPS contained low DNA content and a higher proportion of proteins (Fig. S2). Additionally, dual staining with the Qubit Protein Reagent and Calcofluor White identified three EPS phenotypes: those exhibiting predominantly protein-derived fluorescence, those exhibiting predominantly β-polysaccharide-derived fluorescence, and those exhibiting both (Fig. 4B through D). Fluorescence-based analysis of EPS composition in the ISS strains showed that 74% exhibited protein-derived fluorescence; 11% exhibited β-polysaccharide-derived fluorescence; and approximately 15 % exhibited both (Fig. 4E ). The discrepancy between whole-extract and particle measurements may reflect differences in the sensitivity and specificity of the fluorochromes.

Due to the limited sample volume, whole-extract EPS in PWD water could not be measured quantitatively; thus, only particle analyses were conducted. The fluorescent image analysis of EPS in PWD water showed that 63% of EPS particles displayed protein-derived fluorescence; 31% displayed β-polysaccharide-derived fluorescence; and approximately 6% displayed both (Fig. 4F).

### Characterization of *R. picketti* isolates

**Fig 5:**
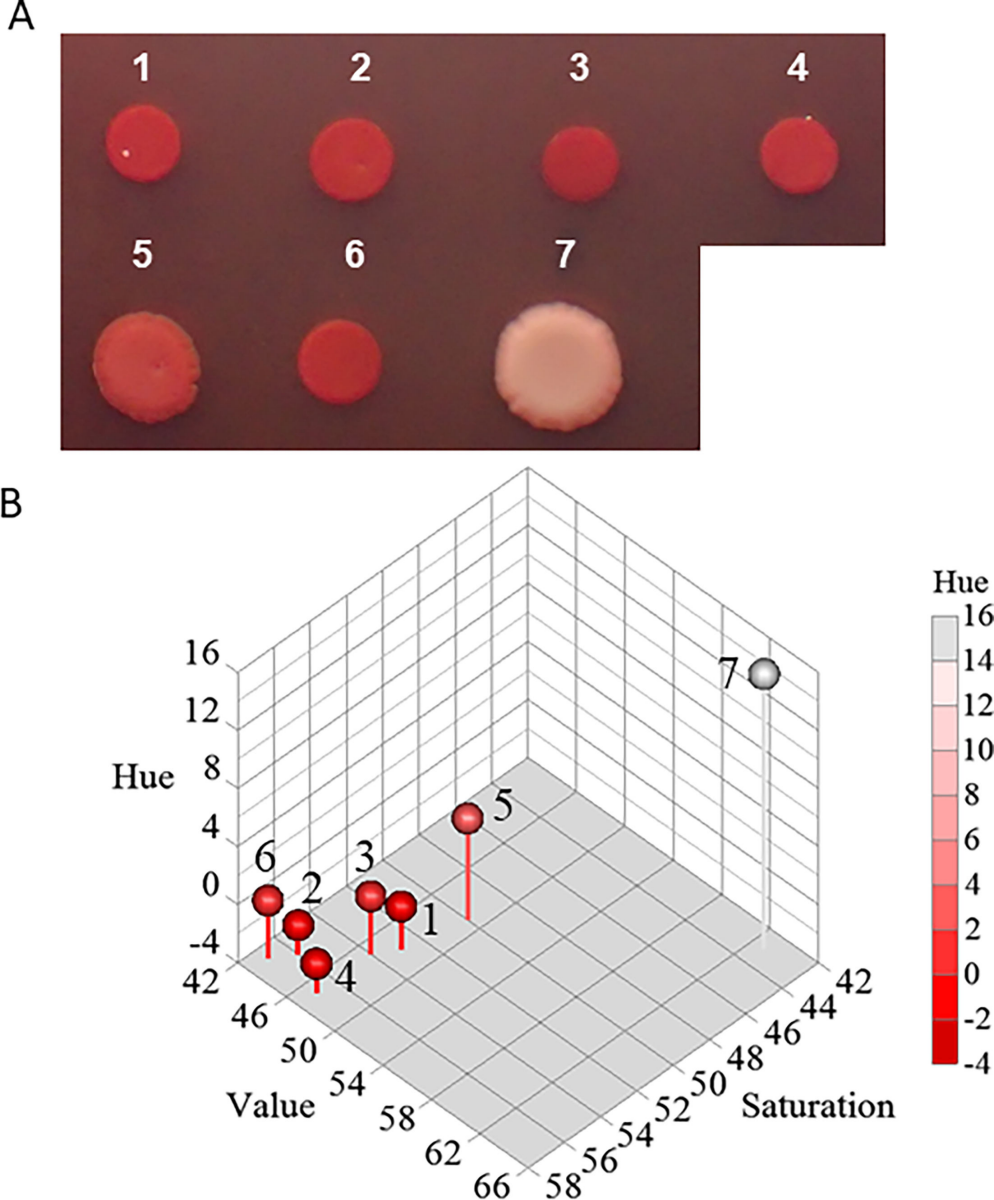
Characteristics of pure cultures of isolates from ISS PWD water. EPS production is indicated with red colonies formed via 100 μg mL^−1^ Congo Red staining on MH agar (**A**). 1, ISS25; 2, ISS26; 3, ISS27; 4, ISS28; 5, NBRC 102503T; 6, NBRC 111592; 7, *E. coli* W3110. Three-dimensional plots of HSV values in the red colonies are shown (**B**). Minus value in the *z* axis represents actual hue value – 360.

It is known that bacteria producing polysaccharides form red colonies on agar media supplemented with Congo Red (6, 7). The *R. pickettii* strains isolated from ISS PWD (ISS25, ISS26, ISS27, and ISS28), along with NBRC 111592, displayed a distinct red color, whereas NBRC 102503T exhibited a slightly lighter red color, and *Escherichia coli* W3110 appeared nearly white with a pink hue (Fig. 5A). These colors exhibited by the colonies could be distinguished from each other based on their hue, saturation, and value (HSV) color values, particularly hue and saturation (Fig. 5B).

**Fig 6:**
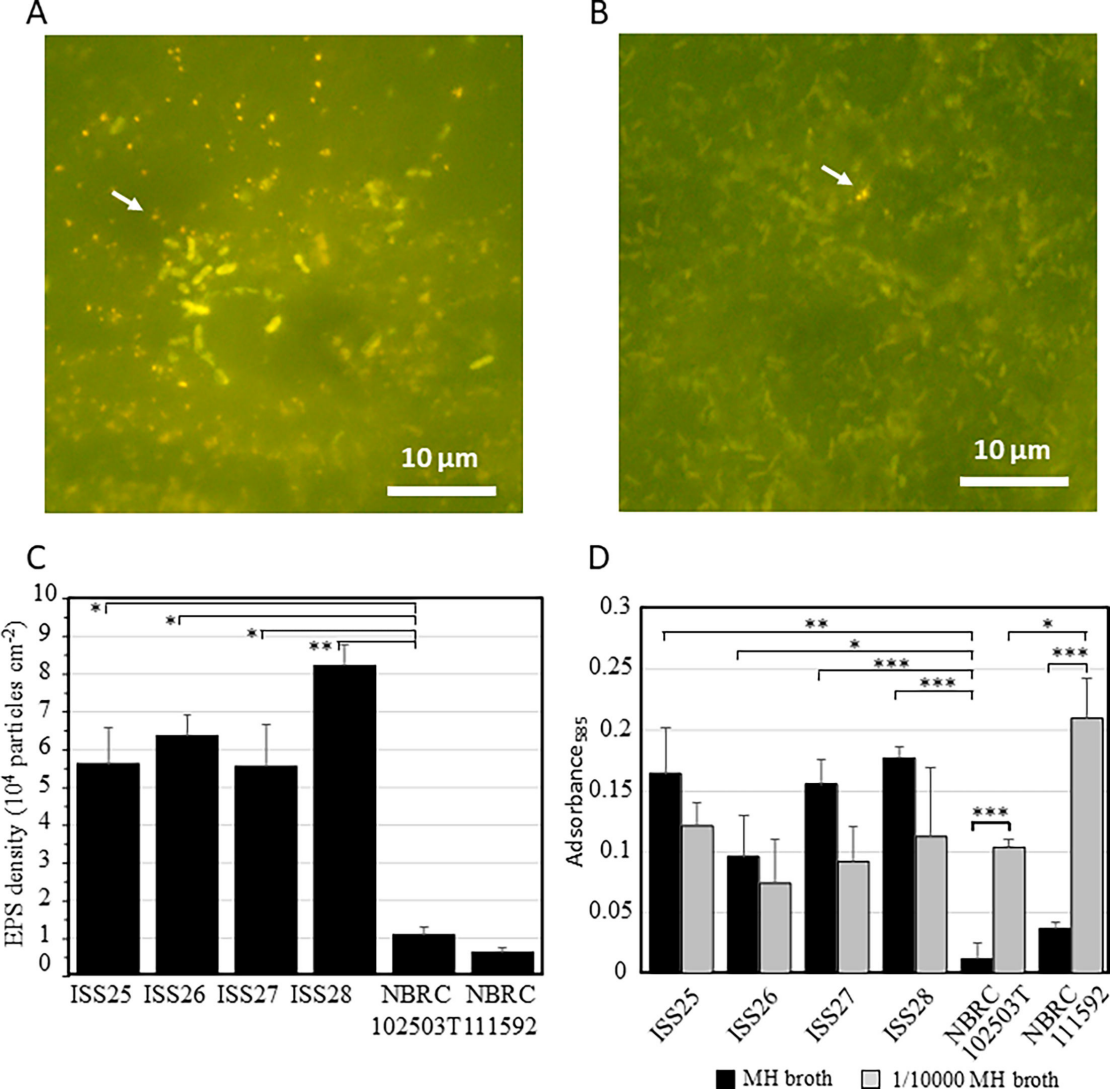
Biofilm formation by *R. pickettii* isolates. Single-species bioﬁlms with ISS25 (**A**) and NBRC 102503T (**B**) were developed statically on nylon 6.6 surfaces. Cells and EPS compounds were labeled with ﬂuorescent dye Qubit Protein Reagent. Arrows indicate typical EPS. EPS density on nylon 6.6 surface is shown as mean ± standard error (*n* = 3) (**C**). Bioﬁlm formation was achieved after 14 days of cultivation, without shaking, of the *R. pickettii* isolates in polystyrene microtiter plate wells containing MH or 1/10,000 broth (**D**). Biofilm amount was determined by quantifying absorbance at 585 nm using NBRC 102503T as a reference after crystal violet staining with the bioﬁlm (*n* = 6). *, *P* < 0.05; **, *P* < 0.01; ***, *P* < 0.001

EPS production is often associated with biofilm-formation ability. As nylon 6.6 is used as a microbial filter material in the PWD unit, we examined EPS attachment on nylon 6.6 sheets and biofilm formation by *R. pickettii* cells (Fig. 6). Abundant EPS particles were observed attached to the sheets in proximity to cells for the ISS strains (Fig. 6A), whereas NBRC 102503T and NBRC 111592 exhibited lower levels of attached and produced EPS (Fig. 6B). All ISS isolates had surface densities exceeding 5 × 10^4^ particles cm^−2^, significantly higher than NBRC 102503T (*P* < 0.05 for ISS25–ISS27 and *P* < 0.01 for ISS28) (Fig. 6C). When evaluated using crystal violet staining, biofilm formation by all ISS isolates was observed to be 8–14 times greater than that by NBRC 102503T (*P* < 0.05 for ISS26, *P* < 0.01 for ISS25, and *P* < 0.001 for ISS27 and ISS28) in MH broth (Fig. 6D). Interestingly, in 1/10,000 MH broth, which has a nutrient-poor condition, NBRC 102503T and NBRC 111592 showed significantly higher biofilm-formation ability than in MH medium (*P* < 0.001), while there was no significant change in the ISS isolate compared to MH medium (*P* > 0.05) and no significant difference between ISS isolates and NBRC 102503T.

## DISCUSSION

*R. pickettii* has been found in the water system and other moist environments within the space station (5, 8, 9). The ISS is a closed environment in which microorganisms brought aboard by items from Earth or by astronauts can proliferate. Water recycling is especially crucial on the ISS, with purification systems utilizing methods such as distillation, ion exchange, particle filtration, and iodine disinfection. However, microorganisms capable of thriving in low-nutrient environments can still proliferate within these systems. In this study, as in our previous work, we found that bacterial concentration in the ISS PWD was about 10^5^ cells mL⁻¹, with *R. pickettii* comprising more than 70% of the bacteria for 3 years, including a previous study (Fig. 1).

This study also found that EPS accounted for 24% and 86% (average 55%) of the total volume concentration of cells in the PWD water (Table 1). Since single EPS particles smaller than 0.2 μm in diameter might pass through the 0.2 μm filter pores, actual EPS biomass may exceed 55% of the estimated total cell biomass. The dominant bacterium in PWD water, *R. pickettii*, produces EPS, and its EPS size and volume under SMG conditions were larger than those under 1G conditions, closely matching the EPS observed in PWD water (Tables 1 and 3). *E. coli* cell volume reportedly increases under SMG condition (10), whereas it is noteworthy that *R. pickettii* cell volume decreases under SMG condition, potentially owing to EPS production; however, the strains exhibited an increase in total volume concentration of cell under SMG condition (Table 2).

EPS produced by bacteria play several essential roles in microbial ecosystems (11, 12). EPS production enables microbial cells to aggregate and form biofilms, creating a protective environment that serves as a barrier against disinfectants and antibiotics (13). Biofilms also shield microorganisms from environmental stresses, including desiccation, ultraviolet light, and heavy metals (14). Additionally, EPS stores essential nutrients for microorganisms and facilitates nutrient transfer (15). It promotes intercellular communication, coordinating collective behavior, which can have several outcomes, including enhanced resistance to antimicrobial agents (16). Within biofilms, EPSs are distributed between cells in a non-uniform pattern (17). EPS molecules interact to form a matrix that encapsulates microbial cells (18).

Generally, EPS consists of polysaccharides, proteins, extracellular DNA, lipids, and other compounds such as humic acids (19). EPSs extracted from *Bacillus subtilis* comprise approximately 62% proteins, 36% carbohydrates, and 1%–2% lipids (20). In an activated sludge culture, EPSs were reported to contain 50.2%, 40.0%, and 9.8% of proteins, polysaccharides, and humic substances, respectively (21). Overall, our results were broadly consistent with these findings (Fig. 4). However, the EPS composition inferred from fluorescent staining in pure culture did not fully match that observed in PWD water. While *R. pickettii* was the predominant species within the bacterial community of PWD water, a variety of other bacterial taxa were also present. Given that bacteria other than *R. pickettii* are capable of producing EPS, it is reasonable to expect differences in both the composition and molecular size of EPS between that produced in a pure culture of *R. pickettii* and those found in PWD water.

In this study, although chemical precipitates and cell debris were removed from the quantification of EPS, there is a possibility that these particles, which are similar in morphology to EPS, were not completely eliminated. In all of the SEM preparation steps, membrane filters with bacterial cells and EPS were kept with the filtration side facing up to avoid contamination. On the other hand, soluble EPS may have been lost during SEM sample preparation. These limitations should be considered when interpreting the results.

Congo Red can react strongly with polysaccharides containing β-(1→4)-linked D-glucopyranosyl units, β-(1→3)-D-glucan, and amyloid proteins (6, 7). Previous studies using Congo Red staining have confirmed that *R. pickettii* isolated from the ISS PWD in 2014 produced EPS (5). This aligns with our findings, as all *R. pickettii* ISS strains isolated from the ISS PWD in this study also produced EPS (Table 3, Fig. 3 to 6). Since Congo Red primarily stains polysaccharides, EPS with a low polysaccharide content may show a weaker red color. Therefore, it is important to evaluate the bacterial EPS production capacity using multiple methods.

Furthermore, we observed that the ability to form biofilms differed between terrestrial and ISS isolates, depending on the nutritional conditions. Terrestrial isolates showed low biofilm-formation ability under nutrient-rich conditions but high ability under nutrient-poor conditions (Fig. 6D). In contrast, ISS isolates exhibited high biofilm-formation ability under both nutrient-rich and nutrient-poor conditions. In addition, the PWD system comprises a sterilizing filter made of nylon 6.6, and we confirmed that *R. pickettii* cells and EPS adhered to the nylon 6.6 sheets, forming biofilms (Fig. 6A and C).

The terrestrial isolates *R. pickettii* NBRC 111592 and NBRC 102503T were isolated from fine bubble water and a patient, respectively. These sources differ substantially from the ISS potable water system, and the resulting differences in habitat and selective pressures likely contributed to the divergence observed between ISS and terrestrial strains. Future work should incorporate more isolates and broader assays. Additionally, the present study was constrained by the limited number of isolates and the small volume of ISS PWD water available. Expanded sampling will be necessary to more fully characterize the microbial ecology of the ISS water environment.

The EPS in PWD water existed as aggregates of multiple EPS particles, and the number of particles in these aggregates was significantly higher than in the pure culture of *R. pickettii* (Fig. 2 and 3). Despite the presence of a sterilizing filter in the PWD, bacterial concentrations in PWD water exceeded 10^5^ cells mL^−1^, along with numerous EPS aggregates (Table 1). Additionally, the ISS strains showed increased total EPS volume concentration under SMG relative to 1G, in contrast to the terrestrial isolate NBRC 111592, which exhibited a decrease under SMG (Table 3). These results suggest that these bacteria adhere to and grow on the nylon 6.6 filters inside the PWD, producing EPS and possibly passing through the filter. The space environment differs from conditions on Earth owing to microgravity, and it has been reported that microorganisms are more prone to forming biofilms in such environments (22). Given that *R. pickettii* forms biofilms, it can easily settle in water-containing equipment, thereby posing a potential long-term problem within the ISS. Drinking water is a unique environment that contains limited organic nutrients. Although this is not ideal for the survival of heterotrophic bacteria, *R. pickettii* demonstrates remarkable adaptability to harsh environments such as drinking water and possesses the ability to thrive.

*R. pickettii* generally does not exhibit pathogenicity in healthy individuals. However, it can cause bacteremia, respiratory infections, and urinary tract infections in immunocompromised patients or those using invasive medical devices (23–27). *R. pickettii* can grow in nutrient-poor environments, such as purified water (28, 29), raising concerns about contamination in the water systems of medical devices and medication solutions. Its propensity to form biofilms complicates cleaning and disinfection efforts, potentially rendering sterilization treatments less effective. *R. pickettii* exhibits adaptive evolution under specific environmental conditions with genetic transmission and sequence diversity (30). Given that *R. pickettii* can infect immunocompromised individuals, there is a potential risk that, as more people inhabit space stations, the PWD could become a source of infection, posing health challenges.

We are attempting to develop a method for assessing microbial load using commercially available biological particle counters (2). This device can quickly and accurately measure the bacterial load in water by selectively detecting biological particles using the fluorescence intensity of riboflavin. Since our ongoing studies have shown that *R. pickettii* can persist in ISS PWD water for extended periods, astronauts need quick and simple methods to monitor bacterial levels and ensure that onboard drinking water remains safe.

*R. pickettii* recovered from the water system of the Mir space station demonstrated significantly higher bacterial counts under SMG than under 1G in high-nutrient media, which is consistent with the results of our study (8). Conversely, under starvation conditions, significantly lower bacterial counts were observed under SMG than at 1G. The response to microgravity appears to depend on the specific strain and nutrient concentration of the medium. In high-nutrient environments, enhanced growth may occur until nutrient availability becomes a limiting factor because of the abundant resources surrounding the cells. Although growth of this bacterium in oligotrophic environments has been documented, the molecular mechanisms underlying its adaptation to these conditions remain largely unknown. Future research is crucial for elucidating the molecular basis of the adaptation of this bacterium to space habitats.

## MATERIALS AND METHODS

### PWD water sampling on the ISS

Potable water sampling on the ISS was conducted three times at 1-year intervals from January 2021 to January 2023, and we used the second (RUN2) and third (RUN3) samples collected on 20 January 2022 and 6 January 2023. An astronaut collected potable water from the PWD on the United States On-orbit Segment of the ISS in NASA’s Post-Flight Analysis Bag. It is made of fluorinated ethylene propylene with polypropylene female Luer lock ports and is used by NASA for water sample collection and analyses. The RUN2 water sample was collected in a 350 mL bag. RUN3 water samples were collected in two bags: one containing 100 mL of potable water and the other containing 250 mL. A 250 mL bag was used in this study. The RUN2 and RUN3 sample bags were loaded into the Cargo Dragon capsules of SpaceX’s CRS-24 and CRS-26 missions, which splashed down into the Gulf of Mexico on 24 January 2022 and 11 January 2023, respectively. The samples were subsequently transported at room temperature to NASA’s Kennedy Space Center (KSC). Upon arrival at the KSC, the bag was stored at 4°C and transported from the KSC to the Tsukuba Space Center, JAXA, Japan, arriving on 2 February 2022 and 23 January 2023, respectively.

### Bacterial community in PWD water

Bacterial cells present in 50 mL of PWD water were trapped onto an autoclaved polycarbonate membrane filter (pore size: 0.2 μm; ADVANTEC, Tokyo, Japan). Bacterial DNA was extracted from the filters using the method described by Ichijo et al. (31) and eluted in 50 μL of TE buffer.

Amplicon sequencing targeting the full length of the 16S rRNA gene was performed using the MinION platform (Oxford Nanopore Technologies, Oxford, UK) equipped with an R9.4.1 Flongle flow cell (Oxford Nanopore Technologies). A 16S rRNA sequencing library was constructed from 10 μL of extracted DNA using the 16S Barcoding Kit 1-24 (Oxford Nanopore Technologies), following the manufacturer’s instructions.

Sequencing was performed using MinKNOW software (Oxford Nanopore Technologies). The resulting FASTQ files were analyzed using the cloud-based EPI2ME FASTQ 16S workflow (Oxford Nanopore Technologies). Reads with a quality score of ≥7 were retained for downstream taxonomic classification.

### Total direct counting of bacteria in PWD water

Total direct counting of bacteria was performed as previously described (2). Bacteria present in the PWD water were filtered through a black polycarbonate filter (pore size, 0.2 µm; ADVANTEC). The filters were then rinsed twice with bacterium-free distilled water. Then, 1 µg mL^−1^ of DAPI in distilled water was applied to the filters and incubated for 3 min at room temperature under dark conditions. The filters were rinsed twice, mounted on glass microscope slides with non-fluorescent immersion oil, and examined under an epifluorescence microscope (DM2500, Leica Microsystems) with an oil immersion objective.

### *R. pickettii* strains

*R. pickettii* strains from the potable water of RUN2 were isolated on R2A agar after incubation at 30°C for 1 week. The four isolates were designated ISS25, ISS26, ISS27, and ISS28. As the reference strain on the ground control, *R. pickettii* NBRC 111592 and NBRC 102503T were obtained from the Biological Resource Center, National Institute of Technology and Evaluation (NBRC), Japan. *R. pickettii* NBRC 111592 was isolated from fine bubble water in 2015. *R. pickettii* NBRC 102503T, deposited at NBRC in 2006, was isolated from a patient who had undergone a tracheotomy. Isolates were grown in MH broth (Becton Dickinson and Co., Franklin Lakes, NJ, USA) at 30°C for 2 days without agitation and stored in 15% glycerol at −80°C until further processing.

### Identification of isolates using the ONT MinION platform

The isolates were identified by sequencing the full-length 16S rRNA gene using MinION equipped with an R9.4.1 flow cell (Oxford Nanopore Technologies) (2). Nearly the full-length 16S rRNA gene was amplified via PCR using primers 16S_8f (5′-AGAGTTTGATCMTGGCTCAG-3′) and 16S_1492r (5′-TACGGYTACCTTGTTACGACTT-3′), supplemented with the universal tails 5′-TTTCTGTTGGTGCTGATATTGC-3′ (forward) and 5′-ACTTGCCTGTCGCTCTATCTTC-3′ (reverse).

Phusion Flash High-Fidelity PCR Master Mix (Thermo Fisher Scientific, Waltham, MA, USA) was used for PCR with the following thermal cycling conditions: 10 s at 98°C; 30 cycles of 5 s at 98°C, 10 s at 55°C, and 30 s at 72°C; and 1 min at 72°C. The PCR products were processed using the SQK-LSK109 kit and barcoded using the EXP-PBC096 kit (Oxford Nanopore Technologies). Library construction was performed according to the manufacturer’s instructions; sequencing was carried out using Oxford Nanopore’s MinKNOW software; and basecalls were performed using Guppy (v.6.0.6) in fast mode. Generated FASTQ files were further analyzed for taxonomic classification using the cloud-based EPI2ME FASTQ 16S workflow with a quality score of ≥8 for quality filtering.

### SEM

Samples (10 mL) from the PWD were filtered through 25 mm diameter polycarbonate membrane filters with a pore size of 0.2 µm (ADVANTEC). Culture isolates (10 µL) were filtered through 25 mm diameter Whatman ANODISC membrane filters with a pore size of 0.02 µm (Global Life Sciences Technologies Japan K.K., Tokyo, Japan). The filters containing bacterial cells and EPSs were placed on a piece of Whatman two filter paper (Global Life Sciences Technologies) soaked in 4% paraformaldehyde and left undisturbed at 4°C for more than 18 h to fix the bacteria. The filters were placed on a piece of filter paper soaked in sterile ultrapure water. For dehydration, the filters were sequentially placed on a piece of filter paper soaked in 50%, 80%, and 100% ethanol and 50%, 80%, and 100% *t*-butyl alcohol at room temperature. In all of the above steps, membrane filters with bacterial cells and EPSs were kept with the filtration side facing up. Polycarbonate filters were cut into eight sections. Each filter section was then transferred to a microtube.

A piece of the polycarbonate filter was placed on a SEM pore (JEOL Datum) connected to a nanopercolator SEM pore holder (JEOL Ltd., Tokyo, Japan). An ANODISC filter was placed on a flat sample holder (JEOL) and incubated with *t*-butyl alcohol. The samples were subsequently freeze-dried and coated with gold. Finally, the gold-coated samples were imaged using a JSM 6510LA scanning electron microscope (JEOL Ltd.) at 15 kV. Electron micrographs were obtained at magnifications of 5,000–30,000 using a secondary electron detector.

Images of bacterial cells and EPS were saved as bmp files, and the length and width of the cells and the diameter of the EPS were analyzed using ImageJ 1.54 J (National Institutes of Health, USA) using the parameters of area and fit ellipse onset measurements. The cell volume (µm^3^) was calculated using the formula *π* × *W*^2^ × (*L* − *W* / 3) / 4, where *W* is the width of the cell (µm), and *L* is the length of the cell (µm) (32).

The EPS volume was calculated using the formula 4 / 3 × *π* × *r*^3^, where *r* is the radius.

The radius and diameter (µm) were determined from the measured area of EPS, where EPS was assumed as a circle using the following formula: area = *π* × *r*^2^.

The length of each pixel was calibrated using a scale in the SEM photograph.

At least 100 cells and EPSs in different fields were measured for quantitative evaluation of size (length and width, diameter) and volume; more than 200 EPSs and more than 700 EPSs in PWD water were used to determine the particle number in EPS aggregates in pure culture, and more than 700 cells and EPSs were counted for determining concentration. The total volume concentration of the cell or EPS (µm^3^ mL^−1^) was determined using the following formula: concentration (mL^−1^) × average vol (µm^3^) of the cell or EPS.

Even in the negative control containing only reagents for SEM preparation, small particles and elongated particle formation were observed on the above filters. The obvious cell debris had an elongated and irregular shape. Therefore, particles exhibiting a length-to-width ratio of less than 1.9 or a diameter exceeding 0.10 µm were measured as EPS. Furthermore, particles that had measurement values similar to EPS, making it impossible to determine whether they were cell debris or EPS, were present at a rate of 1 particle per 120 cells. In the EPS measurement, these were treated as false positives and subtracted from the measured values.

### Growth measurements under SMG

*R. pickettii* strains were cultured in a High Aspect Ratio Vessel (HARV; Synthecon, Houston, TX, USA) system, and growth was monitored by measuring OD_600_, as described by Kim et al. (10). A portion of the frozen culture of *R. pickettii* was inoculated in 1 mL of MH broth and incubated at 30°C overnight. After incubation, 50 µL of the stationary-phase *R. pickettii* culture was inoculated into 50 mL of LB broth. The suspension was then transferred to vessels for HARV incubation. Each vessel was completely filled with the sample and confirmed to be completely free from air bubbles. The cells were positioned either vertically (for incubation under SMG conditions) or horizontally (for incubation under 1G conditions) on an HARV system. The rotation rate was set to 30 rpm to facilitate SMG conditions. The vessels were fitted with a gas-permeable membrane, allowing constant air exchange during incubation. To monitor growth, the HARV system was stopped for approximately 5 min. The reactors for 1G conditions were inverted immediately prior to sampling to homogenize the bacteria. A sterile 1 mL syringe was used to remove 0.125 mL of cell culture through a port on the front plate of the HARV after 18, 24, 36, 48, 72, and 144 h of culture. The cultures were then transferred to sterile cuvettes. The OD_600_ value was aseptically measured using a Biowave CO8000 Cell Density Meter (Biochrom, Ltd., Cambridge, UK). Zero headspace within the HARV was maintained by replacing the removed culture and appropriate amount of MH broth. Each experiment was conducted using four vessels.

### Chemical composition analysis

The EPS preparation protocol was adapted from Jiao et al. (33). *R. pickettii* strains ISS25, ISS26, ISS27, and ISS28 were statically cultured in 200 mL of MH broth at 30°C for 2 days and harvested by centrifugation at 15,000 × *g* for 20 min. Pellets were resuspended in 25 mL of cold PBS, and the biofilm matrix was disrupted using sterile disposable plastic pestles. The cell suspension was stirred at 4°C for 24 h, followed by centrifugation at 15,000 × *g* for 20 min; this step was repeated three times. The resulting supernatant was passed through a 0.2 μm syringe filter, mixed with three volumes of 100% ethanol, and incubated at −20°C for 24 h to precipitate EPS. After centrifugation at 15,000 × *g* for 20 min, the EPS pellets were washed with 70% ethanol and resuspended in 20 mM Tris buffer (pH 8.0). Ethanol precipitation yielded higher EPS recovery than trichloroacetic acid.

DNA concentration in the EPS solution was quantified using the Qubit dsDNA HS Assay Kit (Thermo Fisher Scientific). Protein concentration in the EPS solution was determined with the Qubit Protein Assay Kit (Thermo Fisher Scientific). Total carbohydrate content was measured using the phenol–sulfuric acid-based Total Carbohydrate Assay Kit (Sigma-Aldrich Japan, Tokyo, Japan). All values were expressed per EPS dry weight.

### Fluorescent staining of EPS components and cells

MH broth cultures of individual strains were stained with Qubit Protein Reagent (1 µL in 200 µL Qubit Buffer, protein stain; Thermo Fisher Scientific) for 10 min, DAPI 5 µg mL^−1^ (DNA stain; Nacalai Tesque Inc., Kyoto, Japan) for 10 min, or Fluorescent Brightener 28/Calcoﬂuor White (10 mg mL^−1^, β-polysaccharide stain; Sigma-Aldrich) for 10 min. Stained samples were filtered through 25 mm diameter Whatman ANODISC membrane filters with a pore size of 0.02 µm.

PWD water samples were filtered through 25 mm diameter polycarbonate membrane filters with a pore size of 0.2 µm as described above, and the filter was cut into 16 sections. The filter was dipped in 400 µL of Qubit Protein Buffer, 2 µL of Qubit Protein Reagent, and 5 µg mL^−1^ of DAPI for protein and DNA staining, or 400 µL of Qubit Protein Buffer, 2 µL of Qubit Protein Reagent, and 10 mg mL^−1^ of Fluorescent Brightener 28/Calcoﬂuor White for protein and β-polysaccharide staining.

For biofilm preparation and staining, a 5 × 5 mm square nylon 6.6 sheet was sterilized by ultraviolet irradiation and 70% ethanol, and biofilms were allowed to develop on the sheet in MH broth cultures of individual strains at 30°C for 72 h. For the negative control, the nylon 6.6 sheet without bacterial culture was incubated in MH broth, and no bacterial growth was detected, confirming that sterilization was successfully achieved by the UV treatment. The nylon 6.6 sheets containing pure culture biofilms were subsequently dipped in 400 µL of Qubit Protein Buffer, 2 µL of Qubit Protein Reagent, and 5 µg mL^−1^ of DAPI for 10 min to remove planktonic bacteria and perform fluorescent staining, followed by gentle washing with sterile water.

The ANODISC filter, polycarbonate filter, and nylon 6.6 sheet were mounted in VECTASHIELD PLUS Antifade Mounting Medium (Vector Laboratories Inc., Newark, CA, USA) for observation by epifluorescence microscopy (E-400; Nikon, Tokyo, Japan) with the Nikon filter sets UV-2A for DAPI and Calcofluor White and B-2A for Qubit Protein Reagent. Images were acquired using a CMOS color digital camera (WRAYCAM-VEX830; WRAYMER Inc. Osaka, Japan) and stored as digital files.

### EPS assay

Isolates were individually cultured to stationary phase in MH broth at 30°C for 2 days, and 5 µL each of the cultures was spotted on MH plates containing Congo Red (100 µg mL^−1^, Sigma-Aldrich). The plates were incubated at 30°C for 7 days. Photographs of colonies were captured using an Olympus Stylus TG-4 digital camera (Olympus Co. Tokyo, Japan). The red, green, and blue (*R*, *G*, *B*) values of the colony were calculated using ImageJ 1.54 J (National Institutes of Health) and transformed to HSV values using the following formula (34):

*H* (hue) = 60° × (*G − B*) ÷ [*V* − min(*R*, *G*, *B*)] *V* = *R* in this case

*V* (value [brightness]) = max(*R*, *G*, *B*) ÷ 255

*S* (saturation) = [max(*R*, *G*, *B*) − min(*R*, *G*, *B*)] ÷ max(*R*, *G*, *B*).

### Biofilm-formation assays

Biofilm-formation assays were based on the methodology described by O’Toole and Kolter (35). Overnight liquid cultures of the isolates were transferred to MH broth and grown at 30°C for 2 days until the stationary phase was reached. Then, 5 µL of each culture was used to inoculate the six wells of a 96-well polystyrene microtiter plate (Stem K.K., Tokyo, Japan) containing 195 µL of MH broth for nutrient-rich condition or 1/10,000 MH broth for nutrient-poor condition. Wells containing sterile growth medium were used as negative controls. Plates were incubated at 30°C for 2 weeks without agitation. For biofilm quantification, the culture media and unattached bacterial cells were removed from the wells by careful rinsing with water (three times, 200 µL for each rinse). After drying at 54°C for 30 min, adherent bacteria were stained with 200 µL of a 1% crystal violet solution for 15 min at room temperature. After three gentle rinses with 200 µL of water each, the dye associated with the attached cells was solubilized in 200 µL of 99% ethanol, and the biofilm was quantified by measuring the absorbance of the solution at 585 nm using a microplate reader MTP-810Lab (Corona Electric Co. Ltd., Hitachi, Japan). The absorbance of each sample was calculated by subtracting the absorbance of MH broth alone.

### Statistics

Independent or paired samples *t*-test was performed to compare phenotypic characterization of strains between 1G and SMG condition using the Bell Curve Excel Statistics software (v.3.2; SSRI Co., Ltd., Tokyo, Japan). Dunnett’s method for multiple comparisons was used in ANOVA to compare the means of multiple strain groups to a control group (NBRC 111592).

## Data Availability

The 16S rRNA gene sequences of the isolates generated in this study have been deposited in the DDBJ/ENA/GenBank nucleotide sequence database under accession numbers PV082203–PV082206. The 16S rRNA amplicon sequencing data have been deposited in the DDBJ Sequence Read Archive under accession number DRA021765. All other data supporting the findings of this study are included in this article and its supplemental material.

## SUPPLEMENTAL MATERIAL

Supplemental table and figures spectrum.02913-25-s0001.pdf Table S1, and Figures S1 and S2.

ASM does not own the copyrights to Supplemental Material that may be linked to, or accessed through, an article. The authors have granted ASM a non-exclusive, world-wide license to publish the Supplemental Material files. Please contact the corresponding author directly for reuse.
