# Supplementary material for: High extracellular polymeric substance production and biofilm-forming capacity of Ralstonia pickettii isolates from ISS potable water
Source: Microbiol Spectr. 2026 Feb 5;14(3):e02913-25. doi: 10.1128/spectrum.02913-25 (PMC12955497; doi:10.1128/spectrum.02913-25)
Supplement: Supplemental table and figures — Table S1, and Figures S1 and S2. [file spectrum.02913-25-s0001.pdf]

Table S1. Ratio of EPS to cell biomass in *Ralstonia pickettii* isolates[a]

Component	Gravity and Strain	Number	Diameter	Volume	Total volume
EPS/ Cell	ISS25 1G	2.0	0.034	0.006	0.011
	SMG	1.7	0.045	0.014	0.024
	ISS26 1G	1.6	0.038	0.008	0.014
	SMG	0.94	0.044	0.014	0.013
	ISS27 1G	1.0	0.038	0.007	0.007
	SMG	0.79	0.042	0.010	0.008
	ISS28 1G	1.0	0.039	0.008	0.008
	SMG	0.96	0.049	0.018	0.018
	NBRC[b] 1G	2.7	0.041	0.009	0.023
	SMG	0.48	0.117	0.010	0.005
p-value[c]	Gravity	0.157	0.191	0.022	0.887
	Strain	0.569	0.425	0.485	0.840

[a] Biomass parameters of EPS and cell were derived from Table 2 and Table 3.

[b] *R. pickettii* NBRC 111592

[c] *p*-value in t-test between 1G and SMG or in ANOVA for between strains

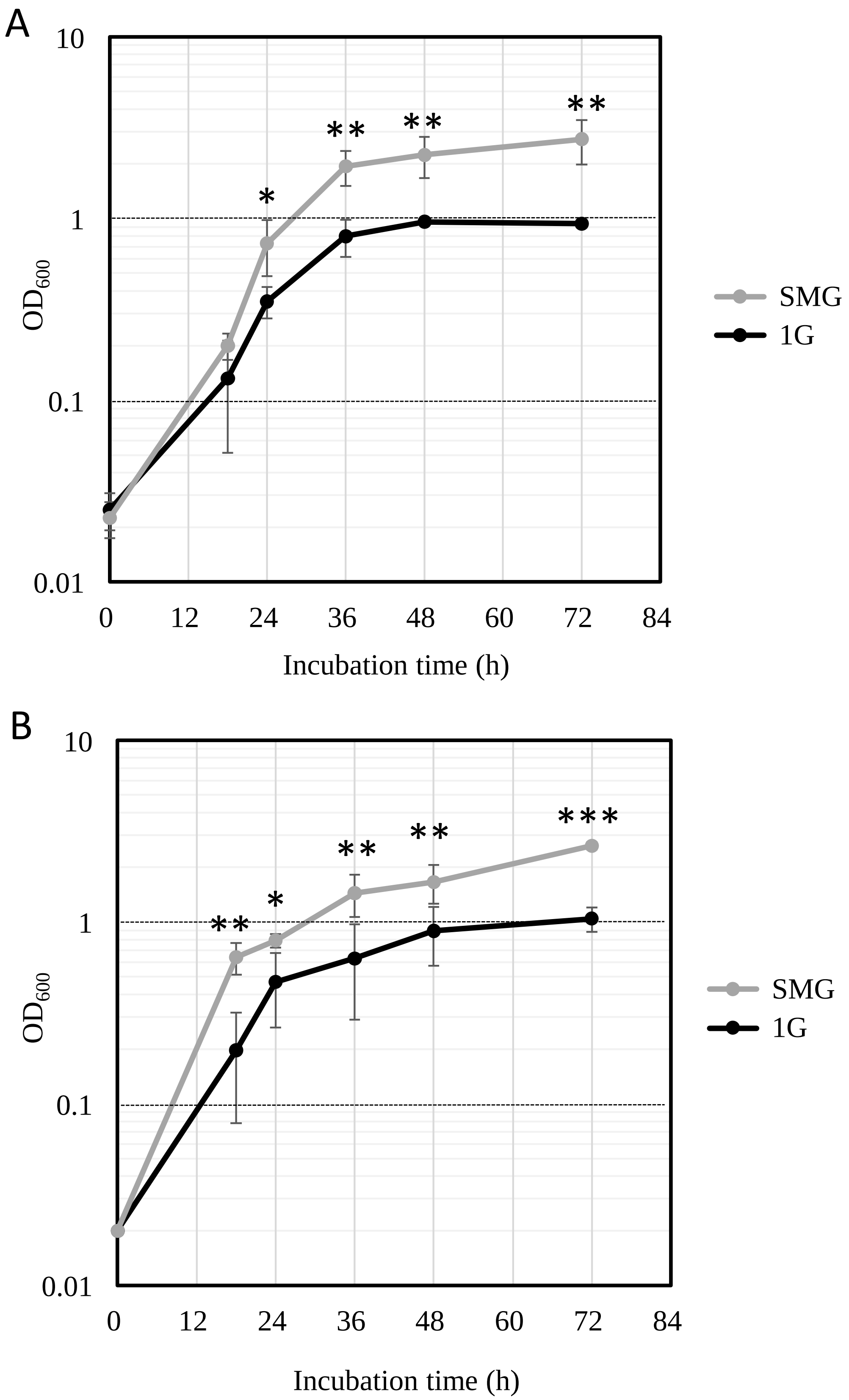

Fig. S1. Growth curve of *R. pickettii* under 1G and SMG conditions.
R. pickettii isolate ISS25 (A) and NBRC111592 were cultured in MH broth.
Values were compared between cultures in 1G and SMG conditions.
*, $p < 0.05$; **, $p < 0.01$; ***, $p < 0.001$.

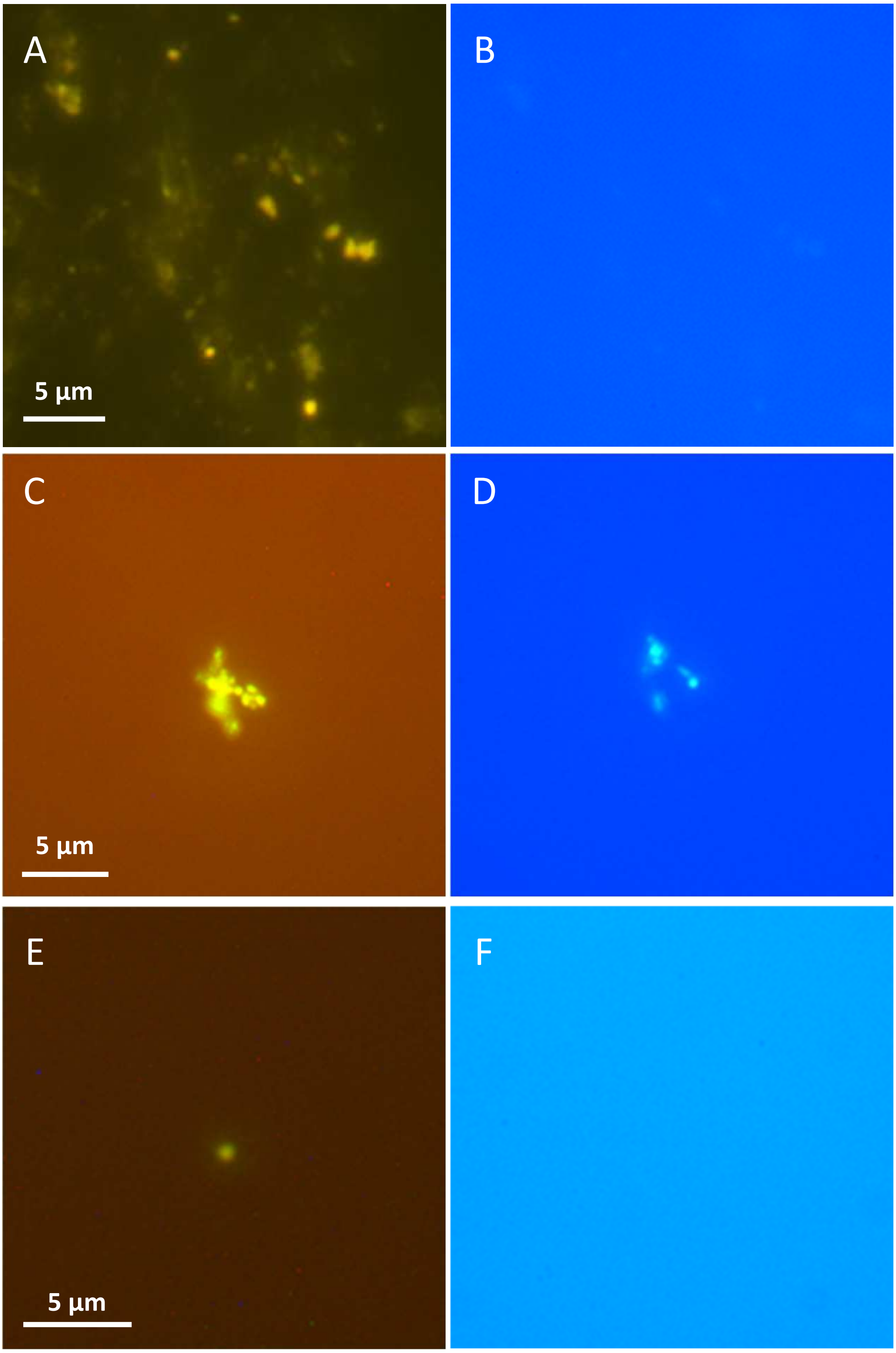

Fig. S2. Fluorescence microscopy images of cells and EPSs in supernatant of the *R. pickettii* ISS25 culture (A and B) and ISS PWD water (C to F). Cells and EPS compounds were labeled by fluorescent dyes Qubit Protein regent (A, C and E) and DAPI (B, D, F).

EPSs showed no DAPI signal because of low or no DNA content. Many EPSs attached to the cells (C and D) and free EPSs (C to F) were noted.
